# Use of Genomic Information in Health Impact Assessment is Yet to Come: A Systematic Review

**DOI:** 10.3390/ijerph17249417

**Published:** 2020-12-15

**Authors:** Balázs Ádám, Szabolcs Lovas, Róza Ádány

**Affiliations:** 1 Department of Public Health and Epidemiology, Faculty of Medicine, University of Debrecen, 4028 Debrecen, Hungary; lovas.szabolcs@med.unideb.hu (S.L.); adany.roza@med.unideb.hu (R.Á.); 2 Institute of Public Health, College of Medicine and Health Sciences, United Arab Emirates University, Al Ain 17666, UAE; 3 Doctoral School of Health Sciences, University of Debrecen, 4028 Debrecen, Hungary; 4 MTA-DE Public Health Research Group, University of Debrecen, 4028 Debrecen, Hungary

**Keywords:** genetic epidemiology, genomics, translation, health impact assessment, public health, public health genomics

## Abstract

Information generated by genetic epidemiology and genomics studies has been accumulating at fast pace, and this knowledge opens new vistas in public health, allowing for the understanding of gene–environment interactions. However, the translation of genome-based knowledge and technologies to the practice of healthcare, and especially of public health, is challenging. Because health impact assessment (HIA) proved to be an effective tool to assist consideration of health issues is sectoral policymaking, this study aimed at exploring its role in the translational process by a systematic literature review on the use of genetic information provided by genetic epidemiology and genomics studies in HIA. PubMed, Scopus, and Web of Science electronic databases were searched and the findings systematically reviewed and reported by the PRISMA guidelines. The review found eight studies that met the inclusion criteria, most of them theoretically discussing the use of HIA for introducing genome-based technologies in healthcare practice, and only two articles considered, in short, the possibility for a generic application of genomic information in HIA. The findings indicate that HIA should be more extensively utilized in the translation of genome-based knowledge to public health practice, and the use of genomic information should be facilitated in the HIA process.

## 1. Introduction

A huge amount of data have already been generated by genetic epidemiology and genomics studies, and the pace of accumulation of genomic information can be expected to accelerate further in the future. The progression of our knowledge about the human genome opens new perspectives in health research, as well as in health promotion and disease prevention in addition to precision medicine. Although the 2005 Bellagio Statement as an international consensus declaration for “the responsible and effective translation of genome-based knowledge and technologies for the benefit of population health” was published 15 years ago, the translation of genomic results to healthcare and public health practice is still cumbersome; therefore, the risk for being ‘lost in transition’ is high [1,2,3,4].

An individual’s health status is determined by the interaction of a multitude of factors, including age, sex, socio-economic conditions, environment, lifestyle, and inherited and acquired genetic make-up [5]. Age, genomic characteristics, and existing medical conditions determine intrinsic vulnerability that can increase the risk of adverse health effects from environmental exposures [6]. Genomics, which is the systematic (genome-wide) study of gene variants and mutations, can detect inherited susceptibility to different diseases as well as mutations induced by environmental exposures, while epigenetics, which is the study of modifications in gene expression rather than in the genetic code itself, is able to identify indicators of early response and also markers of exposure [7]. Owing to these novel scientific approaches, the distinction between genetic and environmental diseases fades away; interlinking information on genome and environment (exposome) becomes possible that can help understanding gene–environment interaction and disease risk comprehensively [8,9,10].

The collection of genomic information by the use of genome-based technologies enables the development of systems biology and, by that, of a truly personalized medicine [11]. Genome-based or genomic technologies include a wide range of techniques used to analyze or apply genomic information with the purpose to understand, prevent, diagnose, and treat disease. These technologies include various -omics using high-throughput sequencing, big-data analysis, genome engineering, and gene therapy [12,13]. As genetic background can influence individual susceptibility in various ways, the highest priority for genomic research should be given to diseases with the strongest evidence of genetic etiology, a high public health impact, and limited ability to modify exposures [4,14,15]. Public health genomics is the area of public health with the task to ensure that results of genomics research (“from cell…”) triggered by innovative technologies are timely, effectively, and responsibly translated to health policies and practice for the benefit of population health (“…to society”) [16]. Khoury et al. defined three priorities for the role of genomics in improving population health: “(1) serving as the honest broker for emerging genomic applications in practice; (2) implementing current evidence-based genomic applications to improve health and prevent disease, while discouraging premature use, misuse, and overuse of genomic applications; and (3) using genomics tools to evaluate the health impact of public health interventions” [8,17]. The task is not easy. Integrative genomics provides a better insight on the pathophysiology of common diseases; however, despite the development of tailored tools, such as the ACCE framework (Analytical validity; Clinical validity; Clinical utility; and Ethical, legal, and social aspects) for the integration of genome-based applications in clinical practice, its use is limited in the prevention and management of diseases [8,18,19,20,21,22,23].

As defined in the Gothenburg consensus paper, “health impact assessment (HIA) is a combination of procedures, methods and tools by which a policy, program or project may be judged as to its potential effects on the health of a population, and the distribution of those effects within the population” [24]. HIA has proved to be an effective method to facilitate consideration of health in sectoral policymaking, thereby supporting the health in all policies approach. As such, HIA may be an appropriate tool to contribute to the translation of genomic information and use of genome-based technologies to healthcare and public health practice. As a member of public health assessment tools (PHATs), HIA can evaluate the health impact of integrating genome-based technologies into healthcare and public health [25]. On the other hand, HIA can also use genomic information in the comprehensive assessment of health impact of any policies, programs, or projects, from the identification of susceptible population groups to susceptibility-specific (stratified) quantitative assessment of health impact. The identification of genetically susceptible population groups is an important aspect of health impact assessment, especially if genetic susceptibility corresponds with socio-economically disadvantaged status. It can be the case with migrants, indigenous people, and ethnic minorities, such as the Roma in several European countries [26]. By revealing genetic susceptibility of certain population groups, assessment of an impact on health inequalities can be performed more precisely. The identification of susceptible groups needs information on the prevalence and populational distribution of genetic variants. In addition, if information on the differing effect size of a risk factor by genetic variants is available, quantitative impact assessment by susceptibility strata becomes feasible.

The aim of this study was to systematically review the use of genomic information originating from genetic epidemiology and genomics studies in health impact assessment.

## 2. Materials and Methods

### 2.1. Research Question

A PICO (population, intervention, comparator, outcome) statement was developed to address and understand the potential use of genomic information in health impact assessment (Table 1).

### 2.2. Identification of Studies

PubMed, Scopus, and Web of Science electronic databases were systematically searched to identify studies from the past three decades assessing, in some way, the use of genomic information in health impact assessment. The search was executed without any restrictions and was conducted using combinations of search terms of “health impact assessment”, “genetic epidemiology”, and “genomics”. The full search strings used in each database are available in the Appendix A. The citations of the search results were imported into the systematic review web application Rayyan [27] and the retrieved studies were screened in two stages by two independent reviewers (Sz.L. and B.A.) based on predetermined inclusion and exclusion criteria.

### 2.3. Assessment of Study Eligibility—Inclusion and Exclusion Criteria

In the first stage, title and abstract of publications were screened, and in the second stage, the full texts of publications selected during the first phase were considered for inclusion. Discrepancies between the judgements of the reviewers regarding the eligibility of studies were remedied by discussion until consensus was reached.

The following inclusion and exclusion criteria were used:

Inclusion criteria: The study

- considers genetic/genomic information of humans acquired by genetic epidemiology or genomics studies,
- discusses the use of genetic/genomic information in health impact assessment,
- is published in peer-reviewed scientific journals, such as research articles, reviews, commentaries, editorials, and citable conference abstracts,
- is published in the previous 30 years (1990–2020),
- is written in English language.

Exclusion criteria: The study

- does not consider genetic/genomic information of humans acquired by genetic epidemiology or genomics studies,
- considers genetic/genomic information of other species but humans,
- does not discuss the use of genetic/genomic information in health impact assessment,
- is published in books, book chapters,
- is not a peer-reviewed publication,
- is published before 1990,
- is published in a language other than English.

Thus, review articles, commentaries/short communications were included but books and book chapters were excluded from the search.

### 2.4. Data Extraction

Two independent reviewers (S.L. and B.A.) carried out the data extraction using Microsoft Excel data extraction sheets that were developed for this study and pilot tested before use. The data extraction included the following variables: publication details, declaration on conflict of interests and funding, study aim and study type, genetic/genomic information (collection, genes, related diseases), health impact assessment (proposal, scope, quantification), use of genetic/genomic information in health impact assessment (advocating of need, discussion of method, direct use), use of health impact assessment to assess the application of genetic/genomic information and/or genome-based technologies in practice (advocating of need, discussion of method, direct use). Discrepancies in extracted data were remedied by discussion until consensus was reached.

Extracted data were descriptively analyzed using Microsoft Excel 2016 (Microsoft Corporation, Redmond, WA, United States). The studies were analyzed from two aspects:

- the use of genetic/genomic information in health impact assessment,
- the use of health impact assessment to assess the application of genetic/genomic information and/or genome-based technologies in practice.

### 2.5. Risk of Bias Assessment

There are several risk of bias assessment tools to be used in the systematic reviews of various topics, such as the Cochrane Collaboration’s tool for randomized controlled trials [28], the Navigation Guide risk of bias assessment domains for environmental health studies [29], the Toxicological data Reliability Assessment Tool (ToxRTool) for toxicological studies [30], the STREGA quality assessment tool for genetic association studies [31]; however, due to the theoretical nature and heterogeneous study design of the finally included studies, existing risk of bias assessment tools could not be applied. The potential existence of bias due to conflict of interest was assessed by the presence of statement on conflict of interest and on funding and by the reviewers’ judgement on any remaining undisclosed conflict of interest.

## 3. Results

### 3.1. Identification of Eligible Studies

**Figure 1:**
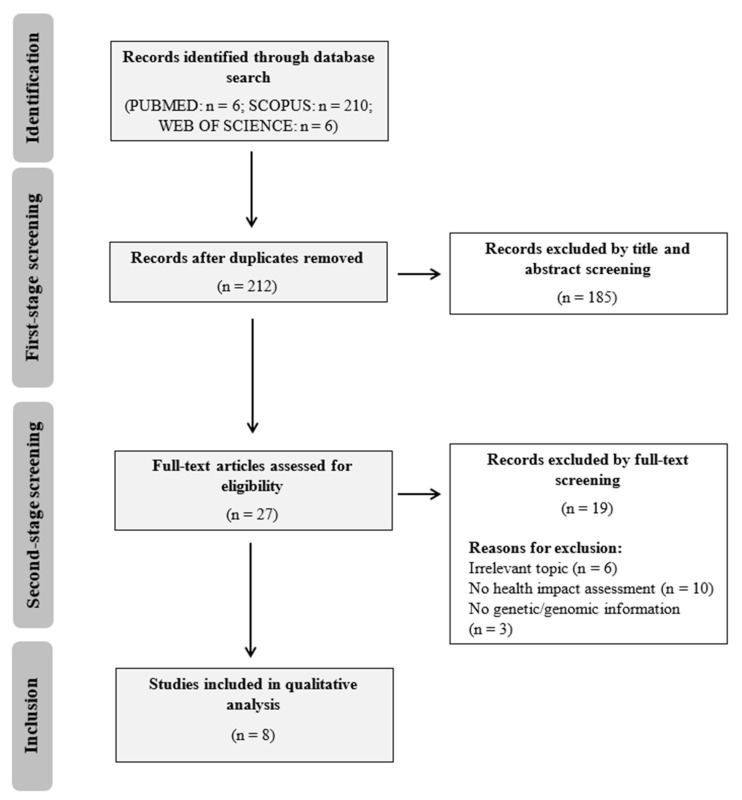
PRISMA flowchart of study selection process.

The process of the search is presented in a PRISMA flowchart (Figure 1). The initial database search yielded 212 studies after removing duplicates. A total of 27 studies were included by the title and abstract screening, and finally, 8 studies met the inclusion criteria after full-text screening.

### 3.2. Summary of Results of Included Studies

The primary findings from the included studies are summarized in Table 2.

The systematic review has not found any publication that reported the conduction of health impact assessment with direct use of genomic information derived from genetic epidemiology or genomics studies. Two of the eight included studies discussed theoretically the use of genomic information in HIA. In a short communication, Smolders et al. reported on the results of the INTARESE/ENVIRISK Workshop “The use of biomarkers for risk assessment” organized in Prague in 2007 [32]. In the perspective of discussing the importance of human biomonitoring, the authors expressed the need for using genomic information in environmental health impact assessment and provided a basic model for an integrated approach that considers lifestyle and person-specific information, which often requires the identification of relevant genetic polymorphisms.

Syurina et al. published a narrative review with the aim to give an overview of existing concepts for the assessment and translation of genome-related innovations to healthcare, applying a descriptive analysis of their present use by public health specialists and policy makers. One of the reviewed eight concepts was HIA, in the description of which the use of genome-based information in the assessment process was mentioned, although the primary consideration of the review was the application of HIA for the translation of genome-based technologies to public health practice. The authors found HIA as an eligible but not systematically used tool for this purpose and presented an example for using HIA to assess the health impact of the expansion of new-born screening [33].

All the remaining six articles discussed only the use of HIA to assess the impact of the application of genomic information and/or genome-based technologies in practice. Rosenkötter et al. published a narrative review of the major assessment instruments in public health (health needs assessment (HNA), health technology assessment (HTA), and health impact assessment), which could contribute to the systematic translation and assessment of genomic health applications. HIA was proposed and found suitable to be used to anticipate consequences that may occur when introducing genome-based technologies for public health purposes. In this way, from the steps that are necessary to translate genomic discoveries to health care and disease prevention, HIA can mainly contribute to the last phase (T4) that seeks to evaluate the ‘real world’ health outcomes of a genomic application in practice on population level, and by that, informs public health policy making [34].

Lal et al. proposed a new theoretical model of valorization to optimize integration and facilitate translation of genome-based technologies to the practice of healthcare systems. The Learning Adapting Leveling (LAL) model is an adapted version of the Basic Design Cycle and the Fish Trap Model and utilizes public health assessment tools that are health needs assessment, health technology assessment, and health impact assessment. HIA is discussed as the final step, assessing the health impact of integrating genome-based technologies into practice, before introducing the concerned technology in the market [35].

In an editorial, Brand discussed the role and challenges of public health genomics to translate the emerging genomic knowledge to public health. As a possible solution to the struggle of all stakeholders, including policy-makers and the private sector, with the translation, she referred to the LAL model and the use of PAHTs, including HIA, in the model [16].

Three more publications from Lal et al. discussed the LAL model too. A 2013 publication deconstructed the LAL model to simplify the steps and by this way to provide improved integration and to facilitate translation of genome-based technologies to the practice of healthcare systems [36]. Lal et al. demonstrated in another publication the overarching reach of the LAL model for translation of genome-based technologies to market and implementation into healthcare systems moving towards personalized healthcare. In both publications, HIA was discussed as a member of PHATs applied in the model. In addition, HIA was recognized to be able to give decision makers insight into the full spectrum of consequences of genome-based technologies or policies and to inform decision making about unpredictability [37]. Finally, HTA from the expanded LAL model was used in a narrative review from Lal et al. that provided a case study to demonstrate the applicability of the model for the translation of host genomic information on the susceptibility to *Chlamydia trachomatis* infection to the healthcare practice of subfertility diagnostics. The study only mentioned HIA as a member of PHATs utilized in the LAL model, but actually HTA was used for the assessment [38].

### 3.3. Risk of Bias Assessment

Two independent reviewers found no risk of bias due to conflict of interest with any of the eight analyzed studies (Table 2).

## 4. Discussion and Recommendations

The systematic review could identify quite few—only eight—studies that dealt with the use of genomic information obtained from genetic epidemiology and genomics studies in health impact assessment. The majority of the studies (seven) considered the use of HIA to assess the application of genomic information and genome-based technologies in practice. Only two of these studies discussed the application of HIA to predict health impact of introducing genome-based technologies for public health purposes of extending newborn screening and assessing the populational health outcomes of genomic applications that can influence public health policy making [33,34]. Most publications presented, from different aspects and at various stages of its development, the Learning Adapting Leveling model, which had been created to facilitate the timely and responsible integration of genome-based information and technologies into the practice of healthcare systems [16,35,36,37,38]. In the model, HIA was applied as a member of public health assessment tools, suitable to serve the last phase of translation (T4—from health practice to health impact).

A major challenge in public health genomics is the translation of research findings to practice. The possible roles of public health professionals and authorities in the facilitation of translational research were identified [8] to be:

- conducting systematic reviews/meta-analyses of reported genetic associations;
- developing evidence-based policy and practice guidelines;
- disseminating, implementing and diffusing research; and
- monitoring population health impact.

HIA has repeatedly been identified as an appropriate method for assessing the future health impact of integrating genomic information and genome-based technologies into practice, being able to provide a forum for stakeholder discussion by its participatory approach, to consider inequality across different population groups and to generate and disseminate valuable information for decision-makers, especially if quantitative assessment is feasible, that is often not otherwise available [7,39,40]. In HIA practice, participatory methods and consideration of inequalities are commonplace, while impact quantification is less frequent [25].

Although HIA has been acknowledged as a suitable tool, its application either alone or as an element of a valorization model is not systematic. The institutionalized systematic use of HIA in the assessment of future health impact of integrating genomic information and genome-based technologies into healthcare, and even more into public health practice, is still a largely unexploited opportunity that needs to be advocated. Although genomic information is accumulating with a growing pace, there is still shortage of genetic susceptibility-specific dose-response coefficients of environmental and lifestyle exposures. Apart from the limited but increasing availability of required data, another problematic barrier is the shortage in experts with solid background in the field of genomics and HIA at the same time. In addition, policymaking processes, into which HIA could be adequately integrated, are heterogeneous and support of policymakers for establishing a systematic application of HIA varies, as well.

The applied search strategy of this review found only two articles that discussed, to a very limited extent, the possibility for a generic application of genomic information in HIA. One of them focused on the use of HIA for the translation of genome-based technologies to practice and only mentioned the possibility to use genome-based information in the assessment process in general [33]. The workshop report from Smolders et al. gave a bit more but still limited explanation to the use of genomic information on personal susceptibility in the integrated approach of environmental health impact assessment [32].

Considering the very little and only theoretical information found in peer-reviewed scientific publications, it can be concluded that the use of genomic information in health impact assessment of topics other than the integration of genome-based technologies into healthcare practice is practically lacking. The observed underuse is in sharp contrast with the enormous perspectives that genomic information can provide in various applications of healthcare and public health practice [41]. In health impact assessment, use of genomic data would allow not only for the precise assessment of future health impacts of policies, programs, and projects by identifying genetically susceptible groups and conducting genetic strata-specific quantitative assessment but for fine-tuning public health interventions to eliminate or at least mitigate the negative and maximize or at least improve the positive effects.

HIA can be considered as a direct route to support decision-making in different nonhealth sectors with targeting not only community health protection/improvement in general but reducing health inequities as well. To identify susceptible population groups that are likely to be affected by policies, programs intended to be introduced is a crucial step in HIA that is mainly based on socio-economic characteristics and sometimes on health conditions of different population groups in the current practice. It is well known that Roma represent the largest and most vulnerable ethnic population in Europe with an estimated number of 10–12 million, and a significant proportion of them live in substandard housing conditions, frequently in segregated colonies [42,43,44,45,46]. In the last decade, public health genomic studies were published to demonstrate that Roma might have increased genetic susceptibility to not only certain rare hereditary diseases but also to metabolic and cardiovascular diseases with very high population prevalence [26]. It was shown that genetic factors contribute to the higher prevalence of reduced HDL-C levels resulting in increased risk to atherosclerosis [47], as well as to that of venous thrombosis [48]; however, no increased genetic susceptibility could be confirmed for diabetes among Roma [49].

The availability of genomic information may not only provide opportunity for a qualitative assessment of health impact stratified by susceptibility but also opens possibility for stratified quantitative analysis. For quantification, information on the strength of association between environmental or lifestyle exposures and health outcomes is required for each group of differing genetic susceptibility. If quantification is carried out by calculating disability-adjusted life years, it entails the need of separate relative risk values that can be quantified by defining unweighted or weighted genetic risk scores for each genetic susceptibility group [50,51], which allows for the calculation of group-specific population attributable risk fractions and finally group-specific attributable disease burden. Such data exist and practice is in place for the sex-specific assessments of the impact of various environmental or lifestyle exposures, and with the accumulation of genomic information, the same will be available for genetic susceptibility-specific analysis in HIA, as well.

## 5. Conclusions

The results of this systematic review clearly demonstrate that the application of genomic information in health impact assessment is still very scarce. The translation of genome-based knowledge and technologies to public health practice is challenging and HIA can be an effective tool in the process. However, the utilization of HIA for this task needs a systematic approach. The use of genomic information in HIA for other purposes than the translation of genome-based knowledge and technologies to practice is a largely unexplored potential that should be promoted to be able to identify susceptible population groups and specifically assess their health impact.

## Figures and Tables

**Table 1:** PICO (population, intervention, comparator, outcome) statement.

PICO Element	Description
Population	Any human subjects whose genomic information has been acquired by genetic epidemiology or genomics studies without restrictions on country, race, religion, sex.
“Intervention”	Genomic information acquired by genetic epidemiology or genomics studies.
Comparator	N/A. There will be no comparator, because only use of genomic information in health impact assessment will be reviewed.
Outcome	Use of genomic information in health impact assessment.

**Table 2:** Characteristics of the selected studies.

Study	Study Type	Use of Genomic Information in HIA		Use of HIA to Assess the Application of Genomic Information/Genome-Based Technologies in Practice		Comments	Conflict of Interest
		Considered?	Way of Consideration	Considered?	Way of Consideration		
Smolders et al., 2010 [32]	Workshop report/short communi-cation	Yes	The need and basic model of using genomic information in environmental health impact assessment was discussed.	No	n.a.	An integrated approach to environmental health impact assessment was proposed, considering lifestyle and person-specific information, which often requires the identification of relevant genetic polymorphisms.	Not detected
Rosenkötter et al., 2010 [34]	Narrative review	No	n.a.	Yes	HIA was proposed to be used to anticipate consequences that may occur when introducing genome-based technologies for public health purposes, which can be supportive of the translational research process.	HIA was found suitable to assess the health impact of introducing genome-based technologies for health care and public health purposes and by that to inform decision makers.	Not detected
Lal et al., 2011 [35]	Theoretical model	No	n.a.	Yes	HIA was discussed as a member of public health assessment tools (PHATs) utilized in the newly developed Learning Adapting Leveling (LAL) model.	A theoretical model of valorization was developed to optimize integration and facilitate translation of genome-based technologies to the practice of healthcare systems. HIA was utilized in the final step, assessing the health impact of integrating genome-based technologies into the practice of healthcare systems.	Not detected
Brand, 2012 [16]	Editorial	No	n.a.	Yes	HIA was discussed as a member of PHATs utilized in the newly developed LAL model.	HIA, as a part of the LAL model, can facilitate the timely and responsible integration of genome-based information and technologies into healthcare systems for the benefit of population health.	Not detected
Syurina et al., 2013 [33]	Narrative review	Yes	Use of genome-based information in HIA was mentioned.	Yes	HIA was identified as an eligible but not systematically used tool for the translation of genome-based technologies to public health.	A theoretical example for the use of HIA on the expansion of new-born screening was presented.	Not detected
Lal et al., 2013a [36]	Theoretical model	No	n.a.	Yes	HIA was discussed as a member of PHATs utilized in the expanded LAL model.	The LAL model was further improved to optimize integration and facilitate translation of genome-based technologies to the practice of healthcare systems. HIA was discussed as the final step, assessing the health impact of integrating genome-based technologies into practice.	Not detected
Lal et al., 2013b [38]	Narrative review, case study	No	n.a.	Yes	HIA was mentioned as a member of PHATs utilized in the LAL model.	HIA was mentioned as an element of the LAL model but health technology assessment was actually used to demonstrate the applicability of the model for the translation of genomic information on the susceptibility to *C. trachomatis* infection to healthcare practice.	Not detected
Lal et al., 2014 [37]	Theoretical model	No	n.a.	Yes	HIA was discussed as a member of PHATs utilized in the LAL model.	The overarching reach of the LAL model for the translation of genome-based technologies to market and implementation into healthcare systems moving towards personalized healthcare was demonstrated. HIA was recognized to be able to give decision makers insight into the full spectrum of consequences of genome-based technologies or policies and to inform them about unpredictability.	Not detected

HIA = Health Impact Assessment; PHAT = Public Health Assessment Tools; LAL = The Learning-Adapting-Leveling model.

## Appendix A

Full search strings by database were:

- PubMed: “health impact assessment”[All Fields] AND ((“genomics”[MeSH Terms] OR “genomics”[All Fields]) OR “genetic epidemiology”[All Fields])
- Scopus: (ALL (“health impact assessment”) AND (ALL (“genetic epidemiology”) OR ALL (genomics))
- Web of Science: ALL FIELDS: (“health impact assessment” AND (“genetic epidemiology” OR genomics))

## References

[1] Burke W., Khoury M.J., Stewart A., Zimmern R.L., Bellagio G. (2006). The path from genome-based research to population health: Development of an international public health genomics network. Genet. Med. Off. J. Am. Coll. Med. Genet..

[2] International Human Genome Sequencing Consortium (2001). Initial sequencing and analysis of the human genome. Nature.

[3] Lenfant C. (2003). Shattuck lecture: Clinical research to clinical practice—Lost in translation?. N. Engl. J. Med..

[4] Lumbreras B., Porta M., Hernandez-Aguado I. (2007). Assessing the social meaning, value and implications of research in genomics. J. Epidemiol. Commun. Health.

[5] Samani N.J., Tomaszewski M., Schunkert H. (2010). The personal genome—The future of personalised medicine?. Lancet.

[6] Solomon G.M., Morello-Frosch R., Zeise L., Faust J.B. (2016). Cumulative Environmental Impacts: Science and Policy to Protect Communities. Annu. Rev. Public Health.

[7] Vineis P., Fecht D. (2018). Environment, cancer and inequalities-The urgent need for prevention. Eur. J. Cancer.

[8] Cleeren E., Van der Heyden J., Brand A., Van Oyen H. (2011). Public health in the genomic era: Will Public Health Genomics contribute to major changes in the prevention of common diseases?. Arch. Public Health Arch. Belg. Sante Publique.

[9] Burton H., Jackson C., Abubakar I. (2014). The impact of genomics on public health practice. Br. Med. Bull..

[10] Budnik L.T., Adam B., Albin M., Banelli B., Baur X., Belpoggi F., Bolognesi C., Broberg K., Gustavsson P., Göen T. (2018). Diagnosis, monitoring and prevention of exposure-related non-communicable diseases in the living and working environment: DiMoPEx-project is designed to determine the impacts of environmental exposure on human health. J. Occup. Med. Toxicol..

[11] Becla L., Lunshof J.E., Gurwitz D., Schulte In den Baumen T., Westerhoff H.V., Lange B.M., Brand A. (2011). Health technology assessment in the era of personalized health care. Int. J. Technol. Assess. Health Care.

[12] Galas D.J., McCormack S.J. (2003). An historical perspective on genomic technologies. Curr. Issues Mol. Biol..

[13] Cunha A. (2017). Genomic technologies—From tools to therapies. Genome Med..

[14] Merikangas K.R., Risch N. (2003). Genomic priorities and public health. Science.

[15] McBride C.M. (2005). Blazing a trail: A public health research agenda in genomics and chronic disease. Prev. Chronic Dis..

[16] Brand A. (2012). Public Health Genomics and personalized healthcare: A pipeline from cell to society. Drug Metab. Drug Interact..

[17] Khoury M.J., Bowen M.S., Burke W., Coates R.J., Dowling N.F., Evans J.P., Reyes M., St Pierre J. (2011). Current priorities for public health practice in addressing the role of human genomics in improving population health. Am. J. Prev. Med..

[18] Boccia S., Mc Kee M., Adany R., Boffetta P., Burton H., Cambon-Thomsen A., Cornel M.C., Gray M., Jani A., Knoppers B.M. (2014). Beyond public health genomics: Proposals from an international working group. Eur. J. Public Health.

[19] Evaluation of Genomic Applications in P., Prevention Working G. (2014). The EGAPP initiative: Lessons learned. Genet. Med. Off. J. Am. Coll. Med Genet..

[20] Djordjevic N., Boccia S., Adany R. (2018). Editorial: Translation of Genomic Results into Public Health Practice. Front. Public Health.

[21] Barna A., Cruz-Sanchez T.M., Brigham K.B., Thuong C.T., Kristensen F.B., Durand-Zaleski I. (2018). Evidence Required by Health Technolgy Assessment and Reimbursement Bodies Evaluating Diagnostic or Prognostic Algorithms That Include Omics Data. Int. J. Technol. Assess. Health Care.

[22] Fiatal S., Adany R. (2017). Application of Single-Nucleotide Polymorphism-Related Risk Estimates in Identification of Increased Genetic Susceptibility to Cardiovascular Diseases: A Literature Review. Front. Public Health.

[23] Boccia S., Pastorino R., Ricciardi W., Adany R., Barnhoorn F., Boffetta P., Cornel M.C., De Vito C., Gray M., Jani A. (2019). How to Integrate Personalized Medicine into Prevention? Recommendations from the Personalized Prevention of Chronic Diseases (PRECeDI) Consortium. Public Health Genom..

[24] European Centre for Health Policy (1999). Health Impact Assessment: Main Concepts and Suggested Approach. Gothenburg Consensus Paper.

[25] Fehr R., Alexanderson K., Favaretti C., de Jong J., La Torre G., Lim T.A., Martin-Olmedo P., Mekel O.C.L., Michelsen K., Rosenkotter N. (2017). Health assessments for health governance-concepts and methodologies. Eur. J. Public Health.

[26] Mendizabal I., Lao O., Marigorta U.M., Kayser M., Comas D. (2013). Implications of population history of European Romani on genetic susceptibility to disease. Hum. Hered..

[27] Ouzzani M., Hammady H., Fedorowicz Z., Elmagarmid A. (2016). Rayyan-a web and mobile app for systematic reviews. Syst. Rev..

[28] Higgins J.P., Altman D.G., Gøtzsche P.C., Jüni P., Moher D., Oxman A.D., Savovic J., Schulz K.F., Weeks L., Sterne J.A. (2011). The Cochrane Collaboration’s tool for assessing risk of bias in randomised trials. BMJ.

[29] Woodruff T.J., Sutton P. (2014). The Navigation Guide systematic review methodology: A rigorous and transparent method for translating environmental health science into better health outcomes. Environ. Health Perspect..

[30] Schneider K., Schwarz M., Burkholder I., Kopp-Schneider A., Edler L., Kinsner-Ovaskainen A., Hartung T., Hoffmann S. (2009). “ToxRTool”, a new tool to assess the reliability of toxicological data. Toxicol. Lett..

[31] Little J., Higgins J.P.T., Ioannidis J.P.A., Moher D., Gagnon F., Von Elm E., Khoury M.J., Cohen B., Davey-Smith G., Grimshaw J. (2009). Strengthening the reporting of genetic association studies (STREGA): An extension of the STROBE statement. Eur. J. Epidemiol..

[32] Smolders R., Bartonova A., Boogaard P.J., Dusinska M., Koppen G., Merlo F., Sram R.J., Vineis P., Schoeters G. (2010). The use of biomarkers for risk assessment: Reporting from the INTARESE/ENVIRISK Workshop in Prague. Int. J. Hyg. Environ. Health.

[33] Syurina E.V., Schulte In den Baumen T., Brand A., Ambrosino E., Feron F.J. (2013). Concepts for the translation of genome-based innovations into public health: A comprehensive overview. Pers. Med..

[34] Rosenkötter N., Vondeling H., Blancquaert I., Mekel O.C., Kristensen F.B., Brand A. (2011). The contribution of health technology assessment, health needs assessment, and health impact assessment to the assessment and translation of technologies in the field of public health genomics. Public Health Genom..

[35] Lal J.A., Schulte In den Baumen T., Morre S.A., Brand A. (2011). Public health and valorization of genome-based technologies: A new model. J. Transl. Med..

[36] Lal J.A., Vaidya A., Gutierrez-Ibarluzea I., Dauben H.P., Brand A. (2013). The Learning-Adapting-Leveling model: From theory to hypothesis of steps for implementation of basic genome-based evidence in personalized medicine. Pers. Med..

[37] Lal J.A., Morre S.A., Brand A. (2014). The overarching framework of translation and integration into healthcare: A case for the LAL model. Pers. Med..

[38] Lal J.A., Malogajski J., Verweij S.P., de Boer P., Ambrosino E., Brand A., Ouburg S., Morre S.A. (2013). Chlamydia trachomatis infections and subfertility: Opportunities to translate host pathogen genomic data into public health. Public Health Genom..

[39] Cole B.L., Shimkhada R., Fielding J.E., Kominski G., Morgenstern H. (2005). Methodologies for realizing the potential of health impact assessment. Am. J. Prev. Med..

[40] Mahboubi P., Parkes M.W., Chan H.M. (2015). Challenges and Opportunities of Integrating Human Health into the Environmental Assessment Process: The Canadian Experience Contextualised to International Efforts. J. Environ. Assess. Policy Manag..

[41] Green E.D., Gunter C., Biesecker L.G., Di Francesco V., Easter C.L., Feingold E.A., Felsenfeld A.L., Kaufman D.J., Ostrander E.A., Pavan W.J. (2020). Strategic vision for improving human health at The Forefront of Genomics. Nat. Cell Biol..

[42] Koupilova I., Epstein H., Holcik J., Hajioff S., McKee M. (2001). Health needs of the Roma population in the Czech and Slovak Republics. Soc. Sci. Med..

[43] Masseria C., Mladovsky P., Hernandez-Quevedo C. (2010). The socio-economic determinants of the health status of Roma in comparison with non-Roma in Bulgaria, Hungary and Romania. Eur. J. Public Health.

[44] Kosa K., Darago L., Adany R. (2011). Environmental survey of segregated habitats of Roma in Hungary: A way to be empowering and reliable in minority research. Eur. J. Public Health.

[45] Fesus G., Ostlin P., McKee M., Adany R. (2012). Policies to improve the health and well-being of Roma people: The European experience. Health Policy.

[46] Council of Europe (2020). Strategic Action Plan for Roma and Traveller Inclusion (2020–2025). https://search.coe.int/cm/Pages/result_details.aspx?ObjectId=0900001680998933.

[47] Piko P., Fiatal S., Kosa Z., Sandor J., Adany R. (2017). Genetic factors exist behind the high prevalence of reduced high-density lipoprotein cholesterol levels in the Roma population. Atherosclerosis.

[48] Fiatal S., Piko P., Kosa Z., Sandor J., Adany R. (2019). Genetic profiling revealed an increased risk of venous thrombosis in the Hungarian Roma population. Thromb. Res..

[49] Werissa N.A., Piko P., Fiatal S., Kosa Z., Sandor J., Adany R. (2019). SNP-Based Genetic Risk Score Modeling Suggests No Increased Genetic Susceptibility of the Roma Population to Type 2 Diabetes Mellitus. Genes.

[50] Talmud P.J., Hingorani A.D., Cooper J.A., Marmot M.G., Brunner E.J., Kumari M., Kivimaki M., Humphries S.E. (2010). Utility of genetic and non-genetic risk factors in prediction of type 2 diabetes: Whitehall II prospective cohort study. BMJ.

[51] Sebastiani P., Solovieff N., Sun J.X. (2012). Naive Bayesian Classifier and Genetic Risk Score for Genetic Risk Prediction of a Categorical Trait: Not so different after all. Front. Genet..

